# The prognostic nutritional index, an independent predictor of overall survival for newly diagnosed follicular lymphoma in China

**DOI:** 10.3389/fnut.2022.981338

**Published:** 2022-10-05

**Authors:** Jingjing Ge, Yaxin Lei, Qing Wen, Yue Zhang, Xiaoshuang Kong, Wenhua Wang, Siyu Qian, Huting Hou, ZeYuan Wang, Shaoxuan Wu, Meng Dong, Mengjie Ding, Xiaolong Wu, Xiaoyan Feng, Linan Zhu, Mingzhi Zhang, Qingjiang Chen, Xudong Zhang

**Affiliations:** Department of Oncology, The First Affiliated Hospital of Zhengzhou University, Zhengzhou, China

**Keywords:** follicular lymphoma, prognostic nutritional index, lymphocyte, albumin, prognosis

## Abstract

**Objective:**

The prognostic nutritional index (PNI) is an important prognostic factor for survival outcomes in various hematological malignancies. The current study focused on exploring the predictive value of the PNI in newly diagnosed follicular lymphoma (FL) in China.

**Materials and methods:**

The clinical indicators and follow-up data of 176 patients who received chemotherapy or immunotherapy combined with chemotherapy with FL in our hospital from January 2016 to March 2022 were retrospectively analyzed. Cox proportional hazard model was used for univariate and multivariate analyses. Kaplan–Meier curves were used to calculate survival rates and draw survival curves. The log-rank test was applied to compare differences between groups.

**Results:**

The optimal cut-off value of PNI was 44.3. All patients were divided into a high PNI group (>44.3) and a low PNI group (≤44.3). The low PNI group had a low CR rate and a high risk of death, with a tendency toward POD24, and Both OS and PFS were worse than those in the high PNI group. PNI was able to predict OS and PFS in FL patients and was the only independent predictor of OS (*P* = *0.014* HR 5.024; 95%CI 1.388∼18.178) in multivariate analysis. PNI could re-stratify patients into groups of high FLIPI score, high FLIPI2 score, no POD24, and rituximab combined with chemotherapy. Moreover, integrating PNI into the FLIPI and FLIPI2 models improved the area under the curve (AUC) for more accurate survival prediction and prognosis.

**Conclusion:**

PNI is a significant prognostic indicator for newly diagnosed FL in China that can early identify patients with poor prognosis and guide clinical treatment decisions.

## Introduction

Follicular lymphoma (FL) is a malignant tumor originating from B cells in the follicular center, it manifests mainly as painless enlargement of lymph nodes at an early stage and may include extrasensory organs at an advanced stage (1, 2). The first-line treatment regimen recommended by guidelines is mainly rituximab (R) combined with chemotherapy, followed by R maintenance therapy after induction therapy, with significant improvement in prognosis (3, 4). The course of follicular lymphoma is usually indolent, and most patients can achieve long-term survival. However, FL has high clinical heterogeneity (5, 6), some patients have a poor response to immunochemotherapy, such as early recurrence and progression, histological transformation (7–9). Identifying high risk patients in a timely manner and improving prognosis have become a current problem and hot research topic (10).

The PNI based on serum albumin and lymphocyte counts is an indicator of nutritional status and systemic inflammation, which can assess nutritional status and surgical risk before surgery (11). Currently, the PNI is widely considered to be related to survival outcomes in malignancy (12, 13). A study showed the PNI to be an independent predictor of OS in elderly patients of FL, with low PNI having poor prognosis (14). There are few relevant studies on PNI in the prognosis of patients with FL, and evidence for its prognostic value is still limited.

In this study, we conducted a single-center retrospective analysis to evaluate the prognostic ability of the PNI in newly diagnosed FL in China. We incorporated it into existing clinical outcome models to improve risk stratification and provide a rationale for achieving precise prognostic stratification and individualized treatment of FL.

## Materials and methods

### Patients

A retrospective analysis of 176 newly diagnosed FL patients admitted to the First Affiliated Hospital of Zhengzhou University from January 2016 to March 2022 was performed. Inclusion criteria: (1) grade 1-3a FL confirmed by pathology and immunohistochemistry (15); (2) not receiving antitumor therapy before diagnosis; (3) complete medical records and follow-up information. Exclusion criteria: (1) diseases with varying effects on lymphocyte levels and albumin levels, such as chronic kidney disease; (2) mixed components or histological transformation in the pathological tissue at diagnosis (16); (3) other tumor-related diseases. This study was approved by The Ethics Committee of Scientific Research and Clinical Trial the First Affiliated Hospital of Zhengzhou University and followed the Declaration of Helsinki.

### Methods

Clinical data of FL patients were collected, such as age, sex, Eastern Cooperative Oncology Group Performance Status (ECOG PS) score, Ann Arbor stage, histological grade, B symptoms, serum albumin, lymphocyte count, hemoglobin (Hb), lactate dehydrogenase (LDH), β2-microglobulin (β2-MG) and bone marrow involvement, Ki-67 expression etc. We analyzed the prognostic PNI in patients with FL. The PNI was calculated as follows: PNI = albumin (g/L) + 5 × lymphocyte count (×10^9^/L) (17).

### Follow-up and endpoints

Follow-up was conducted by consulting hospitalization records and telephone interviews until March 2022, from initial diagnosis to follow-up deadline or date of death. Overall survival (OS) was defined as the time from the date of diagnosis to death or last observation for any cause, Progression-free survival (PFS) was defined as that from the date of diagnosis to first relapse, progression or death and date of the last follow-up (18, 19). Progression of disease within 24 months was defined as POD24 (20). Response assessment was defined as Complete response (CR), Partial Response (PR), Stable disease (SD), and Progressive disease (PD) (21).

### Statistical analysis

SPSS 26.0 software was used for statistical analysis. The Kaplan–Meier method was used to draw survival curves, and the log-rank test was performed in parallel. The optimal cut-off value for stratifying PNI was determined by Receiver operating characteristic curve (ROC) analysis (22, 23). Categorical variables were presented as frequencies and percentages. The chi-square test was used for categorical variables. Cox proportional hazard model was used to analyze univariate association between prognostic factors and OS/PFS (24). All variables with *P* < *0.05* in univariate analysis were retained in multivariate analysis by using forward selection for the best predictor set, and Akaike Information Criteria (AIC) was used to evaluate the model. The PNI was incorporated into the FLIPI and FLIPI2 models, risk grouping was performed again, and the AUC value was used to evaluate the prognostic model. *P* < *0.05* was considered statistically significant.

## Results

### Optimal cut-off point for the prognostic nutritional index

**FIGURE 1:**
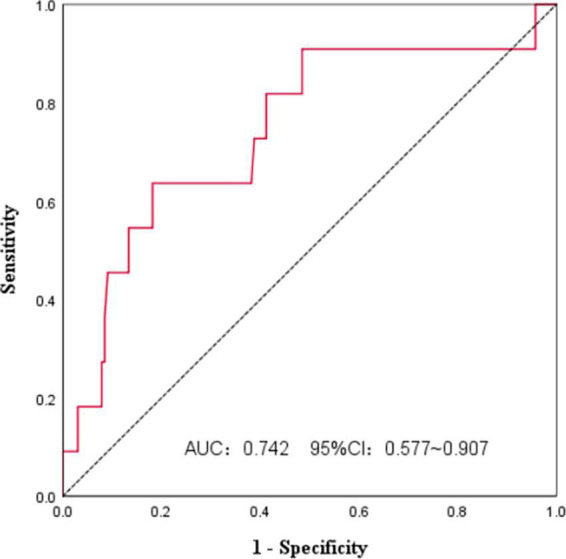
The PNI optimal cut-off point for based on ROC analysis.

We used ROC curves to analyze the PNI and survival of FL patients at initial diagnosis (Figure 1). The AUC was 0.742, and the 95%CI was 0.577∼0.907. The PNI had some accuracy in predicting FL prognosis, and the Jordan index was the largest at a PNI of 44.3, with a sensitivity of 63.6% and specificity of 81.8%. All patients were divided according to the PNI cut-off value into a high PNI group (PNI > 44.3) and a low PNI group (PNI ≤ 44.3).

### Clinical characteristics of patients with follicular lymphoma

Among 176 patients, the median age was approximately 50 (19–85) years old, 49 (27.8%) were >60 years old. Of the patients, 75 (42.6%) were males and 101 (57.4%) females. Most patients were diagnosed with advanced disease. Details were as follows: Ann Arbor Stage III-IV in 138 cases (78.4%); grade 3a in 85 cases (48.3%); ECOG PS ≥ 2 in 30 cases (17.0%); 15 cases (8.5%) with B-cell symptoms; bone marrow participation in 84 cases (47.7%); 47 (26.7%) and 32 (18.2%) cases with LDH and β2-MG exceeding the upper limit of normal; 67 cases (38.1%) with Hb <120 g/L; 123 cases (69.9%) with Ki-67 ≥30%; low, intermediate, and high FLIPI score in 49 (27.8%), 50 (28.4%), and 77 (43.8%) cases; low, intermediate, and high FLIPI2 score in 99 (56.3%), 50 (28.4%) and 27 (15.3%) cases. The high PNI group and the low PNI group had different baseline characteristic distributions in age, ECOG PS score, FLIPI score, FLIPI2 score, Hb, and LDH, and the difference was statistically significant (*P* < *0.05*) (Table 1).

**TABLE 1:** Comparison of patient characteristics between PNI stratifications.

Characteristics	All patients (%)	PNI ≤ 44.3 (%)	PNI > 44.3 (%)	*P*
**Age (median, range)**	50 (19–85)	57 (28–85)	50 (19–82)	**0.027**
≤60	127 (72.2)	22 (57.9)	105 (76.1)	
>60	49 (27.8)	16 (42.1)	33 (23.9)	
**Sex**				0.943
Male	75 (42.6)	16 (42.1)	59 (42.8)	
Female	101 (57.4)	22 (57.9)	79 (57.2)	
**Ann Arbor Stage**				0.154
I/II	38 (21.6)	5 (13.2)	33 (23.9)	
III/IV	138 (78.4)	33 (86.8)	105 (76.1)	
**Histologic grade**				0.088
1–2	91 (51.7)	15 (39.5)	76 (55.1)	
3a	85 (48.3)	23 (60.5)	62 (44.9)	
**ECOG PS**				**0.001**
0–1	146 (83.0)	25 (65.8)	121 (87.7)	
≥2	30 (17.0)	13 (34.2)	17 (12.3)	
**Bone marrow involvement**				0.250
Yes	84 (47.7)	15 (39.5)	69 (50.0)	
No	92 (52.3)	23 (60.5)	69 (50.0)	
**B symptoms**				0.408
Yes	15 (8.5)	5 (13.2)	10 (7.2)	
No	161 (91.5)	33 (86.8)	128 (92.8)	
**Largest lymphnode diameter (cm)**				0.217
≤6	142 (80.7)	28 (73.7)	114 (82.6)	
>6	34 (19.3)	10 (26.3)	24 (17.4)	
**FLIPI score**				**0.001**
Low risk	49 (27.8)	3 (7.9)	46 (33.3)	
Intermediate risk	50 (28.4)	9 (23.7)	41 (29.7)	
High risk	77 (43.8)	26 (68.4)	51 (37.0)	
**FLIPI2 score**				**< 0.001**
Low risk	99 (56.3)	8 (21.1)	91 (65.9)	
Intermediate risk	50 (28.4)	20 (52.6)	30 (21.7)	
High risk	27 (15.3)	10 (26.3)	17 (12.3)	
**PRIMA-PI**				0.125
Low risk	85 (48.3)	19 (50.0)	66 (47.8)	
Intermediate risk	61 (34.7)	9 (23.7)	52 (37.7)	
High risk	30 (17.0)	10 (26.3)	20 (14.5)	
**Hb (g/L)**				**< 0.001**
<120	67 (38.1)	28 (73.7)	39 (28.3)	
≥120	109 (61.9)	10 (26.3)	99 (71.7)	
**LDH (U/L)**				**0.045**
≤245	129 (73.3)	23 (60.5)	106 (76.8)	
>245	47 (26.7)	15 (39.5)	32 (23.2)	
**β 2-MG (mg/L)**				0.052
≤3.0	144 (81.8)	27 (71.1)	117 (84.8)	
>3.0	32 (18.2)	11 (28.9)	21 (15.2)	
**Ki-67**				0.534
<30%	53 (30.1)	13 (34.2)	40 (29.0)	
≥30%	123 (69.9)	25 (65.8)	98 (71.0)	

PNI, Prognostic nutritional index; ECOG PS, Eastern Cooperative Oncology Group Performance Status; FLIPI, Follicular Lymphoma International Prognostic Index; Hb, hemoglobin; LDH, lactate dehydrogenase; β2-MG, β2-microglobulin. The bold values mean that the value is statistically significant.

### First-line treatment options and response to treatment

Treatment initiation was guided by Groupe d’Etude des Lymphomes Folliculaires (GELF) or National Comprehensive Cancer Network (NCCN) criteria. 155 patients (88.1%) received chemotherapy containing rituximab (R), including RCHOP and RCHOP-like regimens and the RCVP, RFC, and BR. 21 patients (11.9%) received chemotherapy alone, and there was no significant difference in the treatment methods between PNI stratifications (*P* > *0.05*). The difference in first-line treatment response between the two groups was statistically significant (*P* = *0.017*). The high PNI group was more likely to achieve CR than the low PNI group, CR rates were 55.8% vs. 34.2%,and the difference in CR rates was statistically significant (*P* = *0.018*). As of the date of follow-up, 38 patients (21.6%) developed POD24, which was more likely to occur in the low PNI group, with an incidence rate of 34.2% vs. 18.1% in the high PNI group (*P* < *0.05*). Six patients underwent re-biopsy at the time of relapse, and pathological findings involved transformation into diffuse large B-cell lymphoma (DLBCL). Histological transformation (HT) was not significantly different between the two groups (Table 2).

**TABLE 2:** Treatment and response data for FL patients.

	All patients (%)	PNI ≤ 44.3 (%)	PNI > 44.3 (%)	*P*
**Treatment**				>0.999
Chemotherapy alone	21 (11.9)	5 (13.2)	16 (11.6)	
Rituximab combine chemotherapy	155 (88.1)	33 (86.8)	122 (88.4)	
**Response to first line treatment**				**0.017**
CR	90 (51.1)	13 (34.2)	77 (55.8)	
PR	32 (18.2)	10 (26.3)	22 (15.9)	
SD	42 (23.9)	9 (23.7)	33 (23.9)	
PD	12 (6.8)	6 (15.8)	6 (4.3)	
**POD24**				**0.033**
Yes	38 (21.6)	13 (34.2)	25 (18.1)	
No	138 (78.4)	25 (65.8)	113 (81.9)	
**HT**				0.611
Yes	6 (3.4)	2 (5.3)	4 (2.9)	
No	170 (96.6)	36 (94.7)	134 (97.1)	

PNI, Prognostic nutritional index; CR, Complete response; PR, Partial response; SD, Stable disease; PD, Progressive disease; POD24, Progression of disease within 24 months; HT, Histological transformation. The bold values mean that the value is statistically significant.

### Univariate analysis of follicular lymphoma patients

Cox regression was used to analyze FL patient results. The results showed that the PNI, ECOG PS score, B symptoms, FLIPI score, and LDH were risk factors for OS, and the PNI, ECOG PS score, and FLIPI score were risk factors for PFS, with a statistically significant difference (*P* < *0.05*). Univariate analysis of meaningful indicators (*P* < *0.05*) was carried out with a stepwise forward method for multivariate analysis. The PNI was the only independent prognostic risk factor for OS in FL patients, and the ECOG PS score was the only independent prognostic risk factor for PFS (Tables 3, 4).

**TABLE 3:** Univariate analysis of patients with FL.

Variables	Factor	OS		PFS	
		HR (95%CI)	*P*	HR (95%CI)	*P*
Age	>60	0.615 (0.133∼2.848)	0.534	1.283 (0.712∼2.312)	0.408
Sex	Male	1.735 (0.529∼5.689)	0.363	1.455 (0.849∼2.495)	0.173
PNI	≤44.3	6.782 (1.982∼23.198)	**0.002**	1.850 (1.014∼3.373)	**0.045**
ECOG PS	≥2	5.539 (1.671∼18.357)	**0.005**	2.456 (1.280∼4.714)	**0.007**
Histologic grade	3a	0.860 (0.262∼2.818)	0.803	1.012 (0.589∼1.738)	0.967
Ann Arbor stage	III/IV	2.979(0.381∼23.283)	0.298	1.381 (0.693∼2.750)	0.358
B symptoms	Yes	4.125 (1.094∼15.553)	**0.036**	1.479 (0.665∼3.290)	0.337
Bone marrow involvement	Yes	0.643 (0.188∼2.198)	0.482	0.916 (0.533∼1.575)	0.752
FLIPI score	≥3	6.588 (1.421∼30.546)	**0.016**	1.902 (1.105∼3.274)	**0.020**
FLIPI-2 score	≥3	3.295 (0.963∼11.268)	0.057	1.586 (0.792∼3.175)	0.193
LDH	>245	3.549 (1.083∼11.635)	**0.037**	1.638 (0.927∼2.895)	0.090
β2-MG	>3	3.064 (0.895∼10.487)	0.074	1.691 (0.864∼3.308)	0.125
Hb	<120	1.962 (0.598∼6.439)	0.266	1.612 (0.934∼2.780)	0.086
Ki-67	≥30%	0.329 (0.100∼1.079)	0.067	1.150 (0.624∼2.120)	0.654

PNI, prognostic nutritional index; ECOG PS, Eastern Cooperative Oncology Group Performance Status; FLIPI, Follicular Lymphoma International Prognostic Index; LDH, lactate dehydrogenase; β2-MG, β2-microglobulin. Hb, Hemoglobin. The bold values mean that the value is statistically significant.

**TABLE 4:** Multivariate analysis of FL patients.

Variables	Factor	OS		PFS	
		HR (95%CI)	*P*	HR (95%CI)	*P*
PNI	≤44.3	5.024 (1.388∼18.178)	**0.014**		NS
ECOG PS score	≥2		NS	2.456 (1.280∼4.714)	**0.007**

PNI, Prognostic nutritional index; ECOG PS, Eastern Cooperative Oncology Group Performance Status; NS, not statistically significant. The bold values mean that the value is statistically significant.

### Overall survival and progression-free survival

**FIGURE 2:**
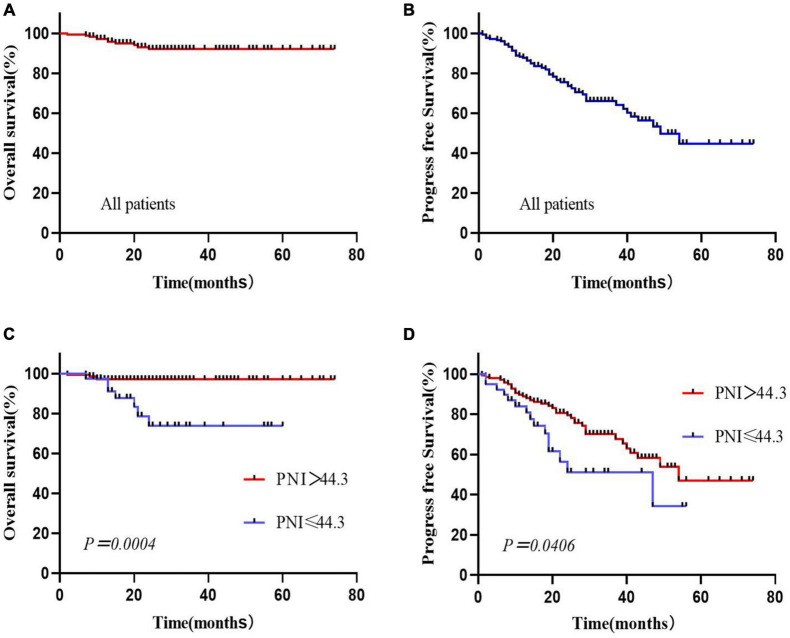
Overall survival and progression-free survival curves for all patients **(A,B)** and stratified by the prognostic nutritional index **(C,D)**.

Among the 176 newly treated FL patients, the median follow-up time was 27 (7–74) months, and 5-year OS and PFS rates were 92.1% and 44.8%, respectively (Figures 2A,B). Kaplan–Meier results showed that OS and PFS were significantly worse in the low PNI group than in the high PNI group (*P* < *0.05*). The 5-year OS rates in the two groups were 73.8% and 97.0%, 5-year PFS in the low PNI group was not reached and the high PNI group was 47.1% (Figures 2C,D). 11 patients (6.3%) died during follow-up with 7 (63.6%) in the low PNI group and 4 (36.4%) in the high PNI group. All died of lymphoma-related comorbidities, with a mortality rate of 18.4% vs. 2.9% in the two groups. Patients with a low PNI had a higher risk of death (*P* = *0.002*).

### Subgroup analysis

**FIGURE 3:**
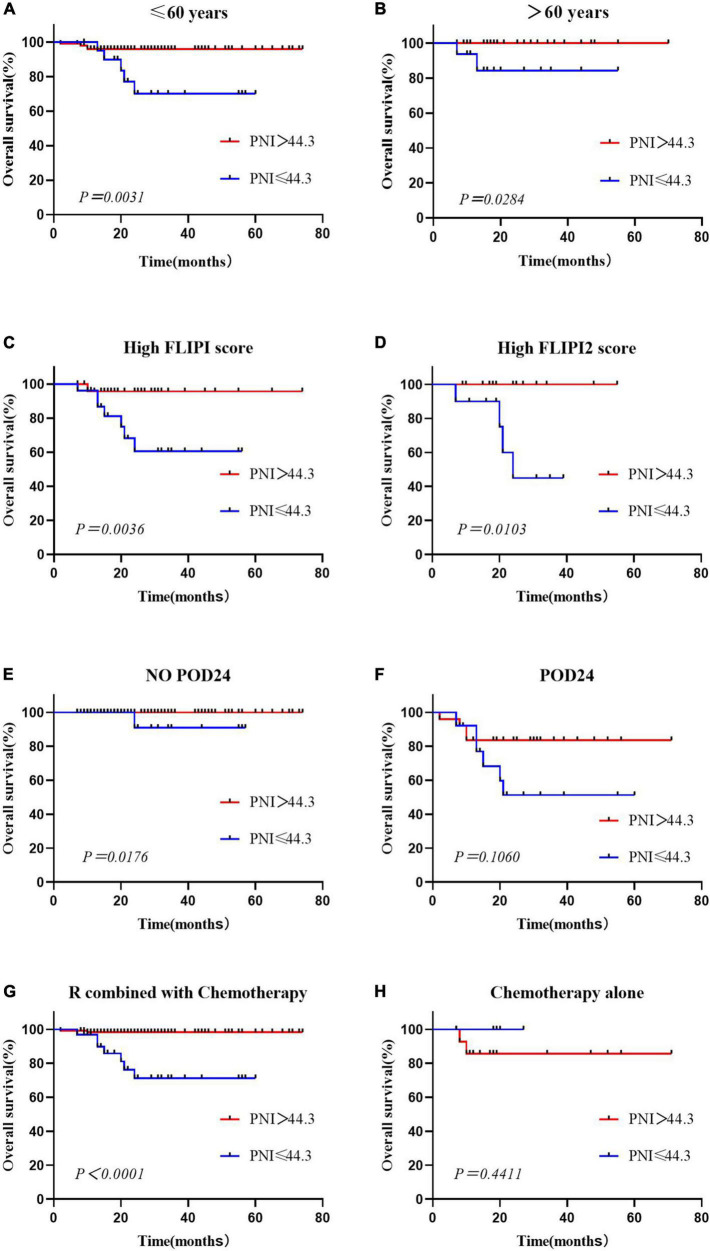
Overall survival according to the prognostic nutritional index and age **(A,B)**, High FLIPI score **(C,D)**, presence or absence of POD24 **(E,F)** and treatment regimen **(G,H)**.

This study showed that PNI was an independent prognostic risk factor for OS in FL patients. Considering the distribution differences between the low and high PNI groups in terms of age, occurrence of POD24, FLIPI score, and first-line treatment regimen, we performed subgroup analysis separately. The results showed that in patients aged ≤60 years and >60 years old, OS of the low PNI group was worse than that of the high PNI group (Figures 3A,B). The PNI was able to stratify the prognosis of patients with high FLIPI scores, and that of patients with low PNI was worse (Figures 3C,D). In the no POD24 group and the R combined with chemotherapy group, patients with low PNI were more likely to have poor prognosis, and there was no difference between the POD24 group and the chemotherapy alone group (Figures 3E–H).

### Prognostic value of prognostic nutritional index

**FIGURE 4:**
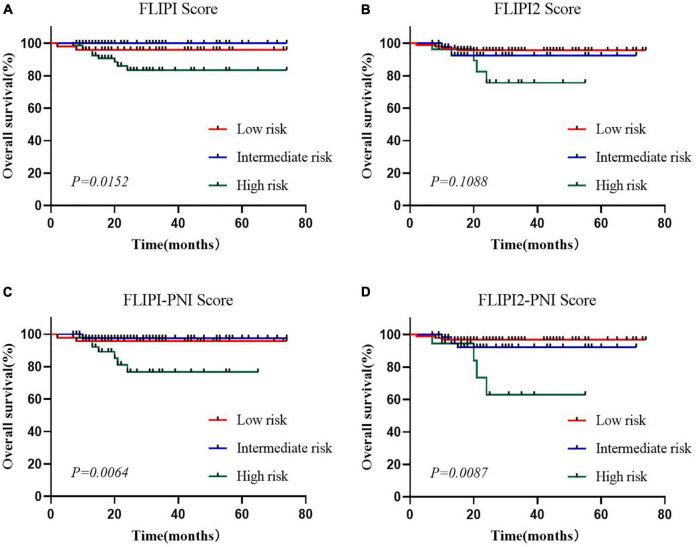
Overall survival for risk groups defined by four scoring systems. **(A)** FLIPI, **(B)** FLIPI2, **(C)** FLIPI-PNI, **(D)** FLIPI2-PNI.

**FIGURE 5:**
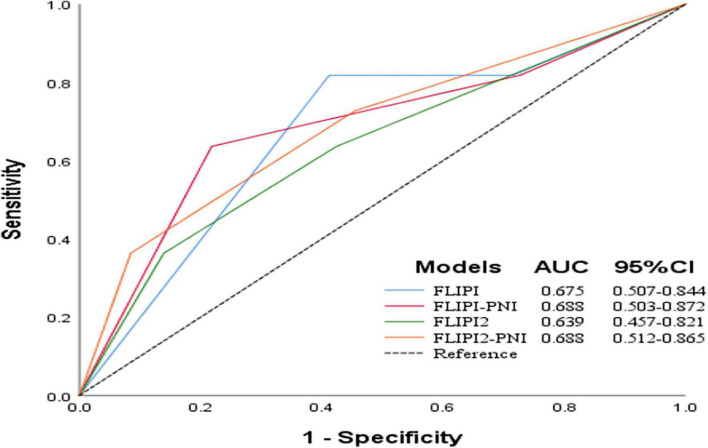
Before and after integrating the PNI with AUC of models for predicting OS.

To evaluate the prognostic ability of the PNI for FL patients, we combined it with the FLIPI score and FLIPI2 score, scoring 1 point for PNI ≤44.3, and then regrouped risk, dividing into three risk categories: low (0–1 points), intermediate (2–3 points), and high (4–6 points) (Table 5). FLIPI-PNI scores were 26.7%, 48.9%, and 24.4% for low, intermediate, and high risks, and FLIPI2-PNI scores were 52.8%, 36.9%, and 10.2%, respectively. Kaplan-Meier curves were generated according to the four scoring systems to estimate the influence of different risk groups on OS (Figures 4A–D). The results showed that in addition to FLIPI2, the prognosis of different risk groups could be distinguished. AUC values for FLIPI-PNI and FLIPI2-PNI were higher than those for FLIPI and FLIPI2 (Figure 5). Incorporating the PNI into the FLIPI score improved AUC and predictive power.

**TABLE 5:** Variables and definitions of different models.

Model and definition	Variable	Point
**FLIPI**	Age (>60 years vs. ≤60 years)	1
Low risk (0–1)	Ann Arbor stage (III–IV vs. I–II)	1
Intermediate risk (2)	Elevated LDH (Yes vs. No)	1
High risk (3–5)	Affected lymph node areas (≥5 vs. <5)	1
	Hb (<120 vs. ≥120)	1
**FLIPI2**	Age (>60 years vs. ≤60 years)	1
Low risk (0–1)	Elevated β2-MG (Yes vs. No)	1
Intermediate risk (2)	Bone marrow involvement (Yes vs. No)	1
High risk (3–5)	Largest lymph node diameter (> 6 vs. ≤6)	1
	Hb (<120 vs. ≥120)	
**FLIPI-PNI**	Age (>60 years vs. ≤60 years)	1
Low risk (0–1)	Ann Arbor stage (III–IV vs. I–II)	1
Intermediate risk (2–3)	Elevated LDH (Yes vs. No)	1
High risk (4–6)	Affected lymph node areas(≥5 vs. <5)	1
	Hb (<120 vs. ≥120)	1
	PNI (PNI ≤ 44.3 vs. PNI > 44.3)	1
**FLIPI2-PNI**	Age (>60 years vs. ≤60 years)	1
Low risk (0–1))	Elevated β2-MG (Yes vs. No)	1
Intermediate risk (2–3)	Bone marrow involvement (Yes vs. No)	1
High risk (4–6)	Largest lymph node diameter (>6 vs. ≤6)	1
	Hb (<120 vs. ≥120)	1
	PNI (PNI ≤ 44.3 vs. PNI > 44.3)	1

FLIPI, Follicular Lymphoma International Prognostic Index; PNI, prognostic nutritional index; Hb, hemoglobin; LDH, lactate dehydrogenase; β2-MG, β2-microglobulin.

## Discussion

Follicular lymphoma (FL) is the most common non-Hodgkin’s lymphoma (NHL) in Western countries, accounting for 22 to 25% of NHL (25, 26), with a median OS of nearly 20 years. However, early progression and HT lead to poor prognosis (27), and there is an urgent clinical need for prognostic indicators that can identify high risk patients. It is well known that the nutritional status of the body can lead to dysfunctional immune function, promoting tumor proliferation and progression. The PNI calculated from the serum albumin level and peripheral blood lymphocyte count is now widely considered to be associated with the prognosis of patients with some solid malignant tumors and hematological malignancies (28–30).

In studies on DLBCL, a low PNI was found to be associated with poor baseline characteristics, lower CR rates, and shorter PFS and OS (31). A study in China showed that the PNI was a reliable prognostic factor for NK/T-cell lymphoma and could improve the performance of commonly used scoring models (32). According to a study in HongKong, PNI was shown to be the independent prognostic factor of PFS in FL and was a cheap and widely available biomarker (19). Some studies have also reported that both the serum albumin level and decreased lymphocyte count were associated with poor OS in FL (33). In this study, we used the peripheral blood index obtained before primary treatment of the disease to calculate the PNI and analyze its prognostic value for FL in primary treatment in China.

The PNI cut-off value in this study was 44.3, compared with the broad cut-off value of 45, which was more accurate for predicting survival, with a sensitivity of 63.6% and specificity of 81.8%. Most patients in the low PNI group had risk factors, such as age >60 years, ECOG PS ≥2, high FLIPI score, LDH higher than normal, and Hb < 120 g/L. There was no significant difference in the treatment regimens between the two groups, but response to first-line treatment was comparable. The high PNI group was more likely to achieve CR, the CR rate was 55.8% vs. 34.2% for the low PNI group. This was consistent with previous studies and indicated that better nutritional status at initial treatment was helpful for achieving CR and providing a theoretical basis for improving nutritional status as soon as possible in FL patients prior to treatment (19).

Studies have shown that approximately 20% of FL patients will experience POD24 with poor survival outcomes, and this was considered a significant adverse prognostic factor for FL (34). A summary analysis of data from 13 randomized clinical trials indicated (35) that male sex [odds ratio (OR), 1.30], PS ≥ 2 (OR,1.63), β2-MG (≥ 3 mg/L; OR,1.43), and high risk FLIPI score (3–5; OR,3.14) were associated with an increased risk of POD24, though no clinical and genetic predictive models were available to accurately predict which patients will experience POD24. In this study, we found a correlation between the PNI and POD24, with a total of 38 patients (21.6%) developing POD24 and a higher incidence in the low PNI group (34.2% vs. 18.1%), showing value for early identification and helping clinicians to develop individual treatment strategies for patients with FL to improve survival.

Multivariate analysis showed that PNI was the only independent factor influencing OS in FL patients. OS and PFS in the low PNI group were significantly worse than those in the high PNI group, and patients with a low PNI had a higher risk of death, which was similar to the results of a retrospective analysis (14). Older FL patients may have lower lymphocyte counts and serum albumin concentrations, leading to a low PNI (36, 37). To avoid attributing the poor prognostic effect in the low PNI group to age factors, we performed stratified survival analysis of patients aged ≤60 years old and >60 years old, and the PNI showed prognostic value for OS in both groups. Moreover, the PNI was able to re-stratify the prognostic risk of the highrisk FLIPI score group, no POD24 group, and R combined with chemotherapy group, with patients with a lower PNI usually having poorer prognosis and shorter survival.

Many prognostic indicators based on clinical data, the tumor microenvironment and gene expression could be used to clinically guide FL-related treatment, such as FLIPI and FLIPI2, which predicted OS and PFS in previously untreated FL patients (38, 39). Nevertheless, early progression could not be accurately predicted. Clinical genomic models such as m7-FLIPI and 23-gene signatures were still difficult to implement clinically due to their cost and complexity (40, 41). Our results showed that the PNI had certain predictive value for FL patients in terms of first-line treatment response, early recurrence and progression risk, and survival prognosis. Adding the PNI to the FLIPI scoring model could improve AUC, with better predictive ability. In the era of immunotherapy, PNI may become an important factor for FL prognosis and risk stratification and has broad clinical application prospects.

The advantage of this study was that patients with diseases affecting nutritional status and other tumors were excluded, all patients were treated in the same treatment institution, and follow-up data were complete, which avoided the influence of confounding factors on prognosis to a certain extent. However, the sample size was relatively limited and was a retrospective study. Conclusions still need to be verified using large-sample prospective data.

In conclusion, the PNI calculation method was simple and easy and could be used as a reliable clinical prognostic index for FL patients. Adding it to the well-established clinical risk model FLIPI improved predictive ability and identify high risk patients early. A large number of prospective and multicenter studies are still needed to confirm this conclusion and provide more useful guidance for individualized treatment of FL.

## Data availability statement

The original contributions presented in this study are included in the article/supplementary material, further inquiries can be directed to the corresponding author.

## Ethics statement

The studies involving human participants were reviewed and approved by the Clinical and Research Ethics Committee of the First Affiliated Hospital of Zhengzhou University. Written informed consent for participation was not required for this study in accordance with the national legislation and the institutional requirements. Written informed consent was not obtained from the individual(s) for the publication of any potentially identifiable images or data included in this article.

## Author contributions

JG, YL, and XZ designed the research. QW, YZ, XK, WW, SQ, HH, ZW, SW, MD, MJD, XW, XF, LZ, MZ, and QC collected and analyzed research data. JG and YL wrote and edited the manuscript. All authors and their respective research teams recruited and followed up the patient, were involved at each stage of manuscript preparation, contributed to the article, and approved the submitted version.
